# CMR Measures of Left Atrial Volume Index and Right Ventricular Function Have Prognostic Value in Chronic Thromboembolic Pulmonary Hypertension

**DOI:** 10.3389/fmed.2022.840196

**Published:** 2022-03-14

**Authors:** Yousef Shahin, Samer Alabed, Syed Rehan Quadery, Robert A. Lewis, Christopher Johns, Dheyaa Alkhanfar, Maria Sukhanenko, Faisal Alandejani, Pankaj Garg, Charlie A. Elliot, Abdul Hameed, Athaniosis Charalampopoulos, James M. Wild, Robin Condliffe, Andrew J. Swift, David G. Kiely

**Affiliations:** ^1^Department of Infection, Immunity and Cardiovascular Disease, University of Sheffield, Sheffield, United Kingdom; ^2^Department of Clinical Radiology, Sheffield Teaching Hospitals NHS FT, Sheffield, United Kingdom; ^3^Sheffield Pulmonary Vascular Disease Unit, Royal Hallamshire Hospital, Sheffield Teaching Hospitals NHS FT, Sheffield, United Kingdom; ^4^INSIGNEO, Institute for in silico Medicine, University of Sheffield, Sheffield, United Kingdom

**Keywords:** cardiac MRI, chronic thromboembolic pulmonary hypertension, left atrium, survival, left heart disease

## Abstract

Providing prognostic information is important when counseling patients and planning treatment strategies in chronic thromboembolic pulmonary hypertension (CTEPH). The aim of this study was to assess the prognostic value of gold standard imaging of cardiac structure and function using cardiac magnetic resonance imaging (CMR) in CTEPH. Consecutive treatment-naive patients with CTEPH who underwent right heart catheterization and CMR between 2011 and 2017 were identified from the ASPIRE (Assessing-the-Specturm-of-Pulmonary-hypertensIon-at-a-REferral-center) registry. CMR metrics were corrected for age and sex where appropriate. Univariate and multivariate regression models were generated to assess the prognostic ability of CMR metrics in CTEPH. Three hundred and seventy-five patients (mean+/-standard deviation: age 64+/-14 years, 49% female) were identified and 181 (48%) had pulmonary endarterectomy (PEA). For all patients with CTEPH, left-ventricular-stroke-volume-index-%predicted (LVSVI%predicted) (*p* = 0.040), left-atrial-volume-index (LAVI) (*p* = 0.030), the presence of comorbidities, incremental shuttle walking test distance (ISWD), mixed venous oxygen saturation and undergoing PEA were independent predictors of mortality at multivariate analysis. In patients undergoing PEA, LAVI (*p* < 0.010), ISWD and comorbidities and in patients not undergoing surgery, right-ventricular-ejection-fraction-%predicted (RVEF%pred) (*p* = 0.040), age and ISWD were independent predictors of mortality. CMR metrics reflecting cardiac function and left heart disease have prognostic value in CTEPH. In those undergoing PEA, LAVI predicts outcome whereas in patients not undergoing PEA RVEF%pred predicts outcome. This study highlights the prognostic value of imaging cardiac structure and function in CTEPH and the importance of considering left heart disease in patients considered for PEA.

## Introduction

Pulmonary hypertension (PH) is heterogeneous and treatment depends on the underlying cause (1). Chronic thromboembolic pulmonary hypertension (CTEPH) is a potentially curable form of PH and a recent meta-analysis has identified a cumulative incidence of 2.9% in patients surviving an acute pulmonary embolism (2). It can also present as PH with no previous evidence of venous thromboembolism (3). It is characterized by non-resolution of thrombus and remodeling of the pulmonary arteries resulting in PH and right ventricular (RV) dysfunction (4) and without treatment a poor prognosis. Pulmonary endarterectomy (PEA), however, provides a potentially curative treatment for selected patients with CTEPH (5) with other options including pulmonary arterial hypertension (PAH) therapies and balloon pulmonary angioplasty (6). Increasingly patients with CTEPH are presenting with comorbidities and additional information that could aid decision making would be helpful.

Several clinical and haemodynamic measurements have been used to assess disease severity and risk stratify patients with PAH. These include an assessment of symptoms (WHO Functional class), exercise capacity (6-min walk test) and measures reflecting RV function including blood based biomarkers (N-terminal pro brain natriuretic peptide) and measures from cardiac catheterization (right atrial pressure, cardiac index and mixed venous oxygen saturation) (7–9). However, some of these measurements are limited by their subjectivity and invasive nature. Nonetheless, a multiparameter risk assessment incorporating a number of these measurements is now recommended in patients with PAH (9) and there is evidence that this approach can also be used in patients with CTEPH (10, 11).

Magnetic resonance imaging using CMR has been shown to have diagnostic value in suspected PH (12–14), prognostic value in PAH (15–17) and in screening for CTEPH using MR perfusion maps (18, 19) and aiding the surgical assessment of CTEPH using MR pulmonary angiography (12, 20). Septal angle, pulmonary artery area and ventricular mass index have additive value in a model to estimate pulmonary artery pressure (14, 21). Measures of RV function including stroke volume, right ventricular end systolic volume and right ventricular ejection fraction predict clinical worsening and mortality and left ventricular measures such as left ventricular end systolic volume predict mortality in PAH (15–17, 22). However, there is limited data on the utility of CMR measures of right ventricular function to aid mortality prediction in CTEPH and to our knowledge no data on the use of left ventricular or atrial measurements.

The aim of this study was, therefore, to assess the prognostic value of gold standard imaging of cardiac structure and function using CMR in a large cohort of patients with CTEPH.

## Materials and Methods

### Patients

Consecutive treatment-naive patients diagnosed with CTEPH who had CMR and right heart catheterization (RHC) up to February 2017 were prospectively recorded in hospital databases as part of the ASPIRE (Assessing the Spectrum of Pulmonary Hypertension Identified at a Referral Center) registry as previously described (23, 24) (ClinicalTrials.gov identifier NCT02565030). Patients' demographics, imaging, and clinical metrics with follow-up data were prospectively collected using a census date of April 29th 2019. CMR and RHC metrics, exercise test, pulmonary function, and treatment were included.

The diagnosis of CTEPH required patients to have undergone RHC with a measured mean pulmonary artery pressure (mPAP) ≥ 25 mmHg at rest and at least one segmental perfusion defect on perfusion lung Q scan or pulmonary artery (PA) obstruction seen by multidetector computed tomography pulmonary angiography (CTPA) or conventional pulmonary angiography with other causes of PH excluded (9).

Following multi-modality imaging patients were discussed at a multi-professional meeting. Patients with CTEPH were subsequently discussed at the national pulmonary endarterectomy MDT at Papworth Hospital, Cambridge where surgical accessibility and suitability for surgery were assessed.

All patients were followed up until the date of death or census date. Ethical approval for this study was granted by our institutional review board (ref c06/Q2308/8). This study was funded by grants from the Wellcome Trust (A.J.S.). The funding body was not involved in the study design or data interpretation.

### Cardiac MRI Acquisition

CMR was performed using an eight channel cardiac coil on a GE HDx (GE Healthcare, Milwaukee, WI) whole body scanner at 1.5 T, as previously described (15). Short-axis cine images were acquired using a cardiac gated multislice balanced SSFP sequence (20 frames per cardiac cycle; slice thickness, 8 mm; field of view, 48 cm; matrix, 2563256; BW, 125 kHz/pixel; TR/TE, 3.7/1.6 ms). A stack of images in the short-axis plane with slice thickness of 8 mm (2-mm inter-slice gap) were acquired fully covering both ventricles from base to apex. End-systole was considered to be the smallest cavity area. End-diastole was defined as the first cine phase of the R-wave triggered acquisition or largest volume. Through plane phase contrast imaging was performed orthogonal to the main pulmonary trunk. Phase contrast imaging parameters were as follows: repetition time, TR 5.6 ms; echo time, TE 2.7 ms; slice thickness, 10 mm; field of view, 48 cm, bandwidth, 62.5 kHz; matrix, 256 3128; 20 reconstructed cardiac phases; and velocity encoding of flow, 150 cm/s. Patients were in the supine position with a surface coil and with retrospective ECG gating.

### Image Analysis

Image analysis was performed on a GE Advantage Workstation 4.1 with the observer blinded to the patient clinical information, and cardiac catheter parameters. Right and left endocardial and epicardial surfaces were manually traced from the stack of short-axis cine images, using proprietary MR workstation software to obtain RV end-diastolic volume (RVEDV) and RV end-systolic volume (RVESV), and left ventricular (LV) end-diastolic volume (LVEDV) and LV end-systolic volume (LVESV). From end-diastolic and end-systolic volumes, RVEF and LV ejection fraction (LVEF) and RV and LV stroke volumes (SV) were calculated. With the exception of RVEF and LVEF, these measurements were all corrected for age and sex (%pred) based on previously published referenced data (25) and they were indexed for body surface area based on Mostellar formula (26). Based on previous work, SV was considered to be the most accurate from LV volumetry (27) and was used for MRI estimation of RV–PA coupling. For calculation of ventricular mass, the interventricular septum was considered as part of the LV. RV end-diastolic mass (RVEDM) and LV end-diastolic mass (LVEDM) were derived. Ventricular mass index (VMI) was defined as RV mass divided by LV mass, as previously described (28). Maximal and minimal PA areas were measured, and relative area change was defined by the following equation: relative area change = (maximum area – minimum area)/minimum area (29). Reproducibility for CMR measurements in our center has been described previously (15).

### Right Heart Catheterization

RHC was performed using a balloon-tipped 7.5F thermodilution catheter (Becton-Dickinson, Franklin Lakes, NJ). RHC was usually performed via the internal jugular vein using a Swan-Ganz catheter. Measurements of right atrial pressure, pulmonary arterial wedge pressure (PAWP), cardiac output (CO) using thermodilution and mixed venous oxygen saturation were also made. Pulmonary vascular resistance (PVR) was calculated as [(mPAP-PAWP) / CO] / 80 and expressed as dyne.s.cm^−5^.

### Pulmonary Arterial Stiffness and Coupling Measurements

As previously described (15, 30–32), RV elastance (Ees) was estimated as mPAP divided by RVESV. PA elastance (Ea) was estimated using mPAP-PAWP divided by LVSV. Ees/Ea by a combined RHC and CMR approach was defined as follows: (mPAP/RVESV)/[(mPAP-PAWP)/LVSV]. CMR estimated Ees/Ea was defined by LVSV/RVESV. Distensibility, a measurement of PA stiffness, was defined as PA relative area change divided by pulse pressure.

### Statistical Analysis

Continuous variables were expressed as mean (SD) for parametric variables and median (interquartile range) for non-parametric variables. Categorical data was presented as the number of subjects and percentage. Continuous variables were compared using independent sample *t*-test. Categorical variables were compared using Pearson's Chi-square test. Survival analysis was conducted using Kaplan-Meier plots and survival was compared using the log-rank test. Survival was calculated from date of diagnosis to date of death or census date to compare survival between the PEA and non-PEA groups. To assess the prognostic value of CMR metrics, survival was calculated from date of CMR to date of death or census date. Univariate Cox proportional hazard regression analysis was used to assess the prognostic value of CMR metrics in terms of biventricular volume, function, mass and PA stiffness metrics in the whole cohort, PEA and non-PEA groups. Hazard ratios (HR) generated from univariate analysis were scaled by dividing the actual individual value of the variable by SD. This is to allow direct comparison of HR. Multivariate Cox proportional hazard regression analysis was performed for predicted CMR variables / MRI coupling measurement, clinical variables and combined CMR and clinical variables. Selected variables were entered into multivariate models if they were reported in literature as predictors of mortality (33, 34), ≤ 10% missing values and a *p* < 0.200 at univariate analysis. Multivariate models for the whole cohort, PEA and non-PEA groups were generated. To overcome multicollinearity, highly correlated variables (*r* > 0.80) were entered separately in the models. Receiver operating characteristics (ROC) analysis was used to assess the prognostic significance of the CMR, clinical and combined CMR and clinical models generated on the whole, PEA and non-PEA cohorts and presented with area under the curve (AUC). In order to evaluate the stability (internal validation) of the CMR prognostic model of the whole cohort, PEA group and non-PEA groups, the bootstrap approach with 1,000 bootstrap samples (default settings) was performed (35). In the bootstrap, simple sampling method with bias-corrected and accelerated 95% confidence interval (95% C.I.) type were selected. Locally estimated scatterplot smoothing regression analysis (LOESS) was performed for 1 year mortality, where significance was demonstrated at multivariate analysis, for patients undergoing and not undergoing PEA. All statistical tests were two-sided and a *p*-value of < 0.050 was considered statistically significant. A Statistical Package for the Social Sciences Program (SPSS) version 26 for Windows (SPSS Inc. Chicago, IL) was used for statistical analysis and for presentation of data GraphPad Prism 8.3.0 (GraphPad Software, San Diego, CA) was used.

## Results

### Baseline Demographics and Measurements

A total of 375 patients (mean+/-SD age 64+/- 14 years, 185 (49%) females) with CTEPH were included in the analysis. One hundred and eighty-one (48%) patients had PEA. Table 1 shows demographic, RHC, and lung function results and Table 2 CMR, RV-PA coupling metrics and pulmonary stiffness data, for the whole cohort and for the PEA and non-PEA groups. The mean+/- SD time delay between RHC and CMR was 45+/-15 days.

**Table 1:** Patients demographics and results of baseline investigations for the whole CTEPH cohort, patients undergoing pulmonary endarterectomy, and not undergoing pulmonary endarterectomy.

**Demographics**	**All patients**	**PEA**	**Non-PEA**	***P*-value**
	**(*n* = 375)**	**(*n* = 181)**	**(*n* = 194)**	
Age, years	64 (14)	60 (14)	67 (13)	<0.001
Female, *n* (%)	185 (49)	88 (23)	97 (26)	0.836
BMI, kg/m^2^	29 (6)	29 (6)	29 (6)	0.354
**Comorbidities**, ***n*** **(%)**				
Malignancy	52 (14)	14 (8)	38 (20)	0.035
CAD	45 (12)	18 (10)	27 (14)	0.237
Left Heart Failure	19 (5)	7 (4)	12 (6)	0.306
CKD	27 (7)	12 (7)	15 (8)	0.680
COPD	35 (9)	13 (7)	22 (11)	0.167
AF	46 (12)	28 (15)	18 (9)	0.068
CVA	26 (7)	17 (9)	9 (5)	0.070
**Right heart catheter metrics**				
mRAP, mmHg	10 (5)	11 (5)	10 (4)	0.618
mPAP, mmHg	44 (12)	44 (12)	44 (13)	0.840
PAWP, mmHg	12 (4)	12 (4)	12 (4)	0.815
PVR, dyne.s.cm^−5^	587 (369)	597 (387)	578 (352)	0.680
CI, L/min/m^2^	2.5 (0.7)	2.5 (0.7)	2.5 (0.7)	0.882
SvO_2_, %	63 (8)	63 (8)	62 (7)	0.337
**Lung function tests and exercise tests**				
FEV1, L	2.17 (0.79)	2.33 (0.77)	2.02 (0.77)	<0.001
FVC, L	3.23 (1.10)	3.41 (1.09)	3.06 (1.07)	0.003
DLCO %pred	57 (20)	59 (21)	55 (18)	0.062
ISWD, m	248 (200)	283 (194)	215 (201)	0.001

*Data is presented as mean (SD) unless otherwise stated*.

*BMI, body mass index; CAD, coronary artery disease; CI, cardiac index; CKD, chronic kidney disease; COPD, chronic obstructive pulmonary disease; CVA, cerebrovascular accident; DLCO, diffusing capacity of the lungs for carbon monoxide; FEV1, forced expiratory volume; FVC, forced vital capacity; ISWD, incremental shuttle walking distance; mPAP, mean pulmonary arterial pressure; mRAP, mean right atrial pressure; PAWP, pulmonary artery wedge pressure; PEA, pulmonary endarterectomy; PVR, pulmonary vascular resistance; SvO_2_, mixed venous oxygen saturation; %pred, percentage predicted*.

**Table 2:** Cardiac MRI imaging parameters for the whole CTEPH cohort, patients undergoing pulmonary endarterectomy, and not undergoing pulmonary endarterectomy.

**Cardiac MR metrics**	**All patients**	**PEA**	**Non-PEA**	***P*-value**
	**(*n* = 375)**	**(*n* = 181)**	**(*n* = 194)**	
RVEDVI %pred	114 (43)	109 (42)	119 (44)	0.021
RVESVI %pred	227 (125)	207 (106)	245 (138)	0.003
RVEF %pred	58 (20)	59 (19)	57 (21)	0.283
RVSVI %pred	63 (25)	63 (27)	64 (24)	0.587
RVEDMI %pred	74 (36)	70 (30)	77 (41)	0.046
LVEDVI %pred	77 (21)	77 (22)	77 (20)	0.995
LVESVI %pred	83 (38)	85 (39)	82 (37)	0.440
LVEF %pred	97 (15)	95 (15)	98 (16)	0.178
LVSVI %pred	74 (23)	74 (23)	75 (23)	0.603
LVEDMI %pred	71 (15)	70 (14)	72 (15)	0.102
LAVI, ml/m^2^	35 (16)	35 (16)	36 (17)	0.710
VMI, %	0.45 (0.21)	0.44 (0.19)	0.46 (0.22)	0.363
**PA stiffness and RV-PA coupling metrics**				
PA RAC, ratio	11 (10)	11 (10)	10 (9)	0.449
PA distensibility, (ΔV/V)/ΔP	0.2 (0.2)	0.2 (0.1)	0.2 (0.1)	0.628
Ees, mmHg/ml/m^2^	1 (0.5)	1 (0.5)	1 (0.5)	0.323
Ea, mmHg/ml/m^2^	1 (0.6)	1 (0.5)	1 (0.6)	0.448
Ees/Ea ratio	2 (1)	2 (1)	2 (1)	0.844
MRI Ees/Ea ratio	0.4 (0.1)	0.4 (0.1)	0.4 (0.1)	0.626

*Data is presented as mean (SD) unless otherwise stated*.

*Ea, arterial load; Ees, right ventricle elastance; LAVI, left atrium volume index; LVEDMI, left ventricle end diastolic mass index; LVEDVI, left ventricle end diastolic volume index; LVEF, left ventricle ejection fraction; LVESVI, left ventricle end systolic volume index; LVSVI, left ventricle stroke volume index; MRI, magnetic resonance imaging; PA, pulmonary artery; PA RAC, pulmonary artery relative area change; RVEDMI, right ventricle end diastolic mass index; RVEDVI, right ventricle end diastolic volume index; RVEF, right ventricle ejection fraction; RVSP, right ventricle systolic pressure; RVESVI, right ventricle end systolic volume index; RVSVI, right ventricle stroke volume index; VMI, ventricular mass index. For other abbreviations see legend for Table 1*.

Compared with the non-PEA group, patients in the PEA group were younger (*p* < 0.001), had a higher incremental shuttle walking distance (ISWD) (*p* = 0.001), lower RVESVI%pred (p=0.003), lower RVEDMI%pred (*p* = 0.046), higher forced expiratory volume at 1 min (FEV1) (*p* < 0.001) and higher forced vital capacity (FVC) (*p* = 0.003).

### Survival Analysis

During the follow up period, 104 (28%) patients died. The median overall survival for the whole cohort was 143 months. Median survival of patients undergoing PEA was higher than patients not undergoing surgery (146 vs. 97 months, 95% C.I. (121–162); *p* < 0.001). Patients' demographics, CMR, RHC, lung function tests and pulmonary arterial stiffness data between survivors and non-survivors is summarized in Supplementary Table 1. Survivors were younger (*p* < 0.001) with a higher percentage of females (*p* < 0.001), had better lung function (*p* < 0.001), exercise capacity and less likely to have a history of malignancy, coronary artery disease, chronic kidney disease or COPD.

### Univariate Analysis of Predictors of Mortality

Univariate Cox proportional hazard regression analysis for CMR metrics and clinical variables for the whole cohort is presented in Supplementary Table 2. Only variables with *p* < 0.200 are shown. CMR measures of RV size and function [RVEDVI%pred (*p* = 0.002), RVESVI%pred (*p* < 0.001), and RVEF%pred (*p* < 0.001)], LV function [LVSVI%pred (*p* = 0.001) and LVEF%pred (*p* = 0.011)], LA volume [LAVI (*p* = 0.010)] and invasive (*p* = 0.027) and non-invasive CMR-derived Ea/Ees ratio (*p* = 0.001), were significant predictors of mortality at univariate Cox regression analysis for the whole cohort. Separate univariate Cox regression analysis was performed for PEA and non-PEA groups and is shown in Tables 3, 4, respectively.

**Table 3:** Univariate Cox proportional hazards regression analysis in patients with CTEPH undergoing pulmonary endarterectomy (metrics shown where *p* < 0.20).

**Covariate**	**Univariate**	**Scaled univariate**	***P*-value**
	**hazard ratio**	**hazard ratio**	
Age, years	1.028 (0.996–1.060)	1.035 (0.998–1.074)	0.087
ISWD, m	0.995 (0.992–0.998)	0.883 (0.871–0.990)	<0.001
**Comorbidities**, ***n***			
Malignancy	3.611 (1.407–9.266)		0.008
CAD	4.577 (1.864–11.241)		0.001
**Cardiac MR metrics**			
LVESVI %pred	1.005 (0.999–1.011)	1.209 (0.953–1.534)	0.117
LVEF %pred	0.980 (0.958–1.001)	0.739 (0.535–1.019)	0.066
LVSVI %pred	0.987 (0.970–1.004)	0.650 (0.425–0.996)	0.135
LAVI, ml/m^2^	1.029 (1.015–1.044)	1.513 (1.173–1.952)	<0.001
**Right heart catheter metrics**			
SvO_2_, %	0.932 (0.883–0.984)	0.598 (0.403–0.887)	0.011
**Lung function tests**			
FEV1 %pred	0.463 (0.261–0.823)	0.641 (0.498–0.825)	0.009
DLCO %pred	0.660 (0.500–0.873)	0.479 (0.361–0.635)	0.004

*Data in paracenteses is 95% confidence interval*.

*For abbreviations see legend for Tables 1, 2*.

**Table 4:** Univariate Cox proportional hazards regression analysis in patients with CTEPH not undergoing pulmonary endarterectomy (metrics shown where *p* < 0.20).

**Covariate**	**Univariate**	**Scaled univariate**	***P*-value**
	**hazard ratio**	**hazard ratio**	
Age, years	1.032 (1.011–1.054)	1.044 (1.020–1.062)	0.003
ISWD, m	0.997 (0.996–0.999)	0.799 (0.774–0.883)	0.001
**Comorbidities**, ***n***			
Malignancy	1.747 (1.030–2.962)		0.038
CAD	2.455 (1.425–4.227)		0.001
COPD	1.692 (0.932–3.070)		0.087
Left Heart Failure	2.064 (0.944–4.514)		0.070
**Cardiac MR metrics**			
RVEDVI %pred	1.007 (1.002–1.011)	1.338 (1.095–1.634)	0.004
RVESVI %pred	1.002 (1.001–1.004)	1.322 (1.119–1.560)	0.001
RVEF %pred	0.983 (0.973–0.993)	0.706 (0.574–0.870)	0.001
RVEDMI %pred	1.005 (1.000–1.009)	1.192 (1.012–1.403)	0.035
LVEDVI %pred	0.988 (0.977–1.000)	0.792 (0.632–0.993)	0.043
LVEF %pred	0.984 (0.971–0.998)	0.789 (0.641–0.969)	0.010
LVSVI %pred	0.984 (0.974–0.994)	0.689 (0.547–0.867)	0.002
LVEDMI %pred	1.010 (0.995–1.024)	1.150 (0.930–1.423)	0.197
VMI, %	2.110 (0.869–5.110)	2.300 (0.996–5.220)	0.099
**Right heart catheter metrics**			
mPAP, mmHg	1.022 (1.003–1.041)	1.295 (1.035–1.609)	0.024
mRAP, mmHg	1.087 (1.041–1.136)	1.521 (1.221–1.893)	<0.001
CO, L/min	0.790 (0.663–0.942)	0.702 (0.540–0.914)	0.009
PVR, dyne.s.cm^−5^	1.001 (1.000–1.002)	1.536 (1.190–1.981)	0.001
SvO_2_, %	0.914 (0.888–0.941)	0.519 (0.421–0.639)	<0.001
**Lung function tests**			
FEV1 %pred	0.678 (0.472–0.973)	0.735 (0.552–0.979)	0.035
DLCO %pred	0.719 (0.609–0.850)	0.534 (0.389–0.734)	<0.001
**PA stiffness and RV-PA coupling metrics**			
Ees, mmHg/ml/m^2^	0.552 (0.308–0.998)	0.756 (0.575–0.995)	0.046
Ea, mmHg/ml/m^2^	1.341 (0.991–1.816)	1.196 (0.994–1.439)	0.057
Ees/Ea ratio	0.637 (0.475–0.855)	0.649 (0.472–0.893)	0.003
MRI Ees/Ea ratio	0.095 (0.021–0.443)	0.719 (0.582–0.889)	0.002

*Data in paracenteses is 95% confidence interval*.

*For abbreviations see legend for Tables 1, 2*.

### Multivariate Analysis of Predictors of Mortality

Multivariate Cox proportional hazard regression analysis models of only CMR metrics, clinical variables and CMR metrics and clinical variables (*p* < 0.200) at univariate analysis are presented in Table 5. In the whole cohort, higher RVESVI%pred (*p* = 0.007), lower LVSVI%pred (*p* = 0.001) and higher LAVI (*p* = 0.001) were independent predictors of increased mortality in the CMR metrics model. The regression equation for the multivariate Cox regression model in the whole cohort is as follows: Expected hazard = (RVESV%pred^*^0.002)-(LVSVI%pred^*^0.019) + (LAVI^*^0.013). In the PEA group, lower LVSVI%pred (*p* = 0.035) and higher LAVI (*p* < 0.001) (Expected hazard = (LAVI^*^0.030) - (LVSVI%pred^*^0.018) and in the non-PEA group, lower RVEF%pred (*p* = 0.015) and lower LVSVI%pred (*p* = 0.024) were independent predictors of increased mortality [Expected hazard = (RVEF%pred^*^-0.167) - (LVSVI%pred^*^0.014)]. In the combined model, lower LVSVI%pred (*p* = 0.040) and higher LAVI (*p* = 0.033) in the whole cohort, higher LAVI (*p* = 0.009) in the PEA group and lower RVEF%pred (*p* = 0.040) in the non-PEA group were predictors of increased mortality.

**Table 5:** Multivariate cardiac MR metrics, clinical variables, and combined cardiac MR and clinical variables Cox regression model in the whole CTEPH cohort, endarterectomy and non-endarterectomy groups.

**Covariate**	**Multivariate hazard ratio**	***P*-value**
**Cardiac MR model**		
**Whole cohort**		
RVESVI %pred	1.002 (1.001–1.003)	0.007
LVSVI %pred	0.984 (0.974–0.993)	0.001
LAVI, ml/m^2^	1.013 (1.005–1.020)	0.001
**PEA group**		
LVSVI %pred	0.982 (0.966–0.999)	0.035
LAVI, ml/m^2^	1.030 (1.017–1.044)	<0.001
**Non-PEA group**		
RVEF %pred	0.846 (0.739–0.968)	0.015
LVSVI %pred	0.986 (0.974–0.998)	0.024
**Clinical model**		
**Whole cohort**		
ISWD, m	0.997 (0.995–0.999)	0.001
PEA, *n*	0.384 (0.231–0.631)	<0.001
CAD, *n*	2.357 (1.398–3.973)	0.001
COPD, *n*	1.915 (1.056–3.472)	0.032
Malignancy, *n*	2.513 (1.454–4.433)	0.001
SvO_2_, %	0.946 (0.917–0.977)	0.001
**PEA group**		
ISWD, m	0.995 (0.991–0.998)	0.004
CAD, *n*	3.543 (1.331–9.431)	0.011
Malignancy, *n*	2.897 (1.008–8.328)	0.042
**Non-PEA group**		
ISWD, m	0.998 (0.996–1.000)	0.015
CAD, *n*	1.956 (1.087–3.520)	0.025
Malignancy, *n*	1.948 (1.117–3.396)	0.019
mRAP, mmHg	1.094 (1.040–1.149)	<0.001
**Combined cardiac MR and clinical model**		
**Whole cohort**		
ISWD, m	0.997 (0.995–0.999)	0.002
PEA, *n*	0.467 (0.271–0.804)	0.006
CAD, *n*	2.605 (1.497–4.532)	0.001
Malignancy, *n*	1.952 (1.059–3.595)	0.032
CKD, *n*	2.092 (1.056–4.144)	0.034
SvO_2_, %	0.953 (0.918–0.990)	0.013
LVSVI %pred	0.960 (0.924–0.998)	0.040
LAVI, ml/m^2^	1.015 (1.001–1.028)	0.033
**PEA group**		
ISWD, m	0.993 (0.988–0.997)	<0.001
LAVI, ml/m^2^	1.022 (1.020–1.032)	0.043
Malignancy, *n*	3.034 (1.043–8.823)	0.042
CAD, *n*	4.063 (1.681–9.817)	0.002
**Non-PEA group**		
Age, years	1.044 (1.013–1.075)	0.004
ISWD, m	0.998 (0.995–1.000)	0.024
RVEF %pred	0.961 (0.931–0.999)	0.017
RVEDVI %pred	1.025 (1.001–1.029)	0.039

*Data in paracenteses is 95% confidence interval*.

*For abbreviations see legend for Tables 1, 2*.

**Figure 1:**
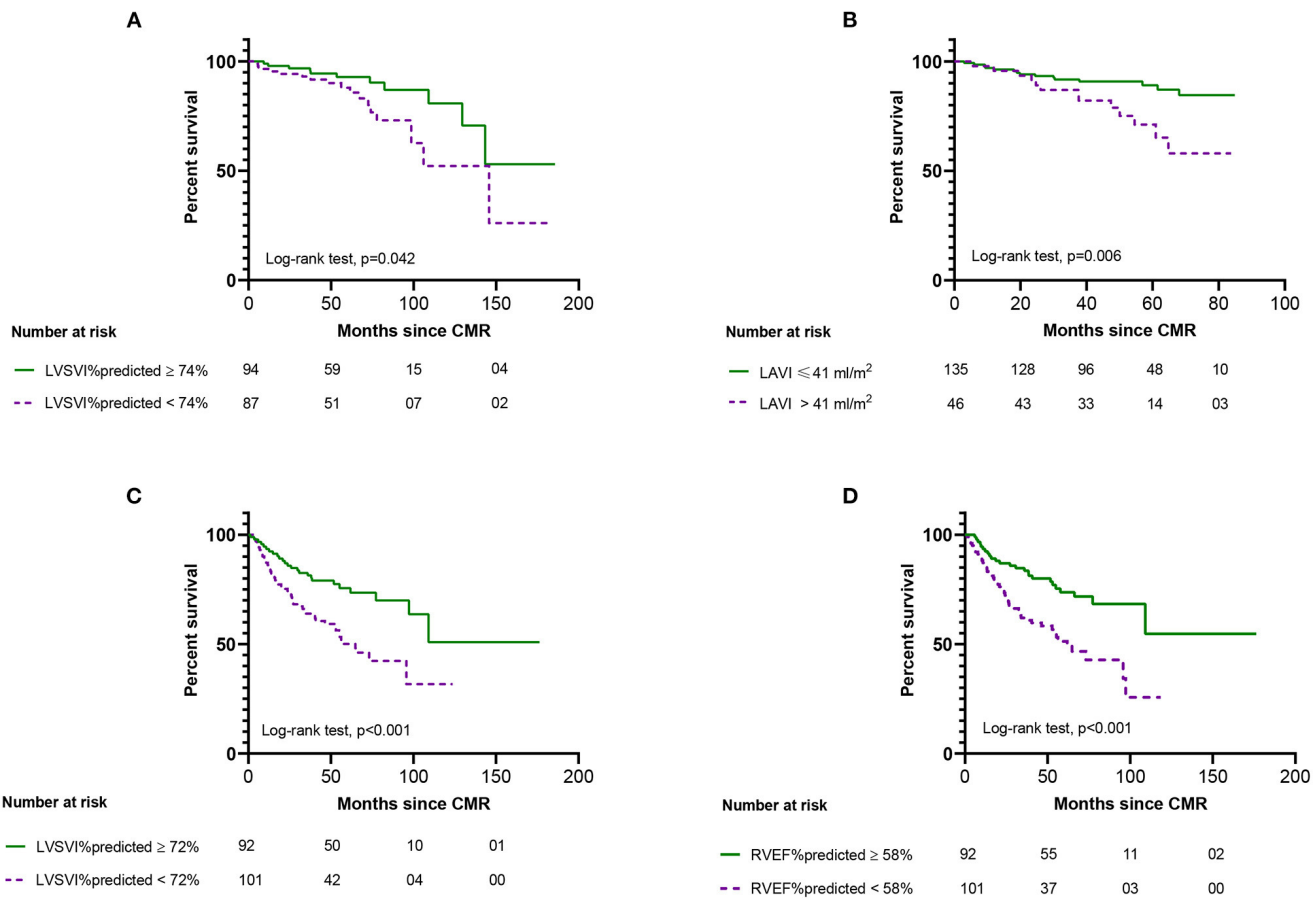
Kaplan-Meier survival analysis from date of cardiac MR showing the outcome of patients undergoing pulmonary endarterectomy (top) based on left ventricular stroke volume index %predicted **(A)** and left atrial volume index **(B)** and for patients not undergoing pulmonary endarterectomy (bottom) based on left ventricular stroke volume index %predicted **(C)** and right ventricular ejection fraction %predicted **(D)**. Numbers at risk for each group are presented below the plot.

**Figure 2:**
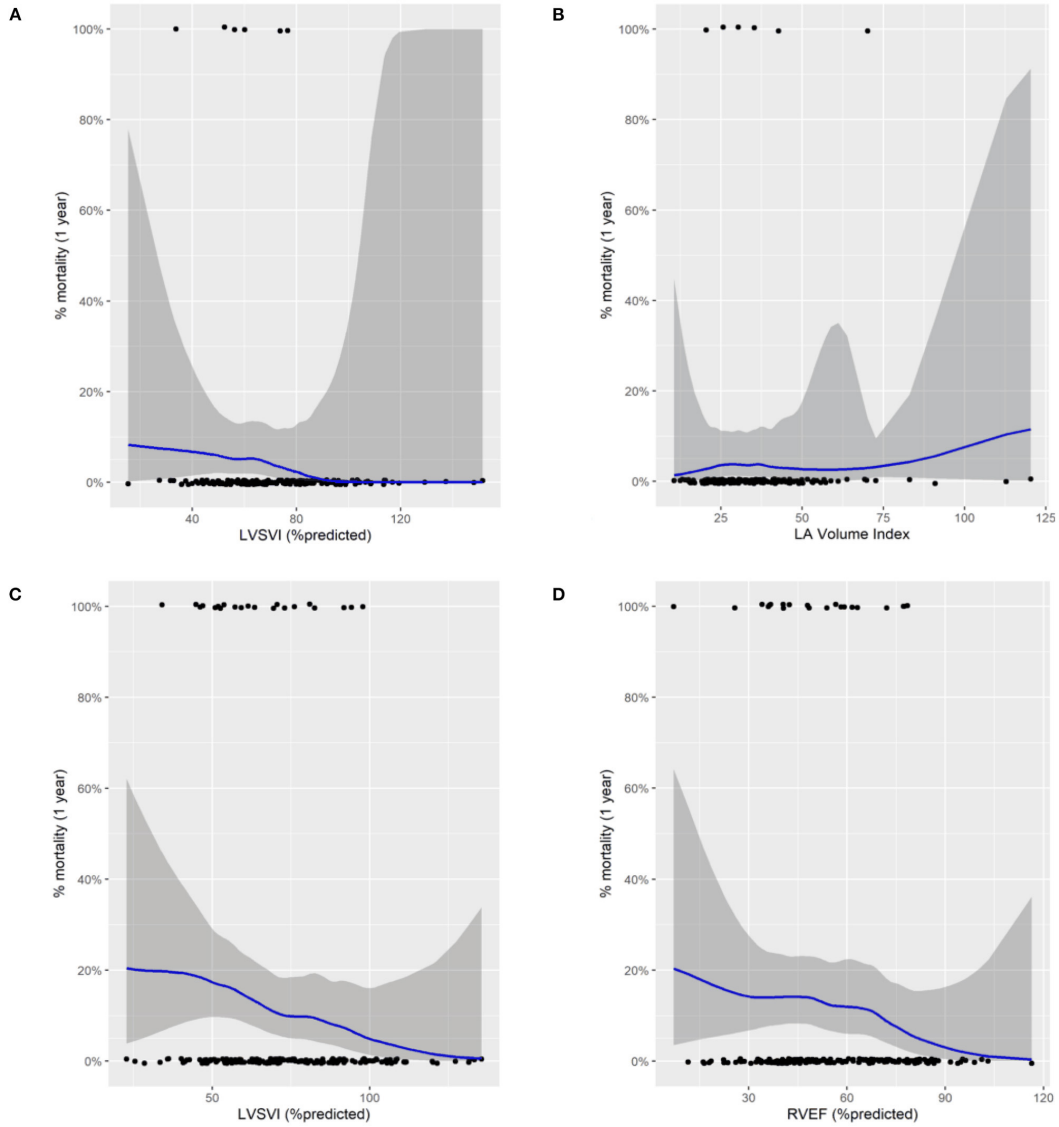
Locally estimated scatterplot smoothing regression analysis (LOESS) for risk of 1 year mortality for patients undergoing pulmonary endarterectomy (top) for left ventricular stroke volume index %predicted **(A)** and left atrial volume index **(B)** and patients not undergoing pulmonary endarterectomy for left ventricular stroke volume index %predicted **(C)** and for right ventricular ejection fraction %predicted **(D)**. Black dots represent status (alive at the bottom and dead at the top). The blue line correspond to a smoothing function fitted by loess regression.

Figure 1 shows Kaplan-Meier survival analysis of the PEA group (LVSVI%pred and LAVI) and non-PEA group (LVSVI%pred and RVEF%pred). LAVI is plotted above and below a value of 41 ml/m^2^ based on previous work and for other values based on median (36). Figure 2 shows LOESS regression analysis for risk of 1 year mortality for LVSVI%pred and LAVI for patients undergoing PEA and for LVSVI%pred and RVEF%pred for the Non-PEA group.

### Accuracy, Sensitivity, and Specificity of Prognostic Models

**Figure 3:**
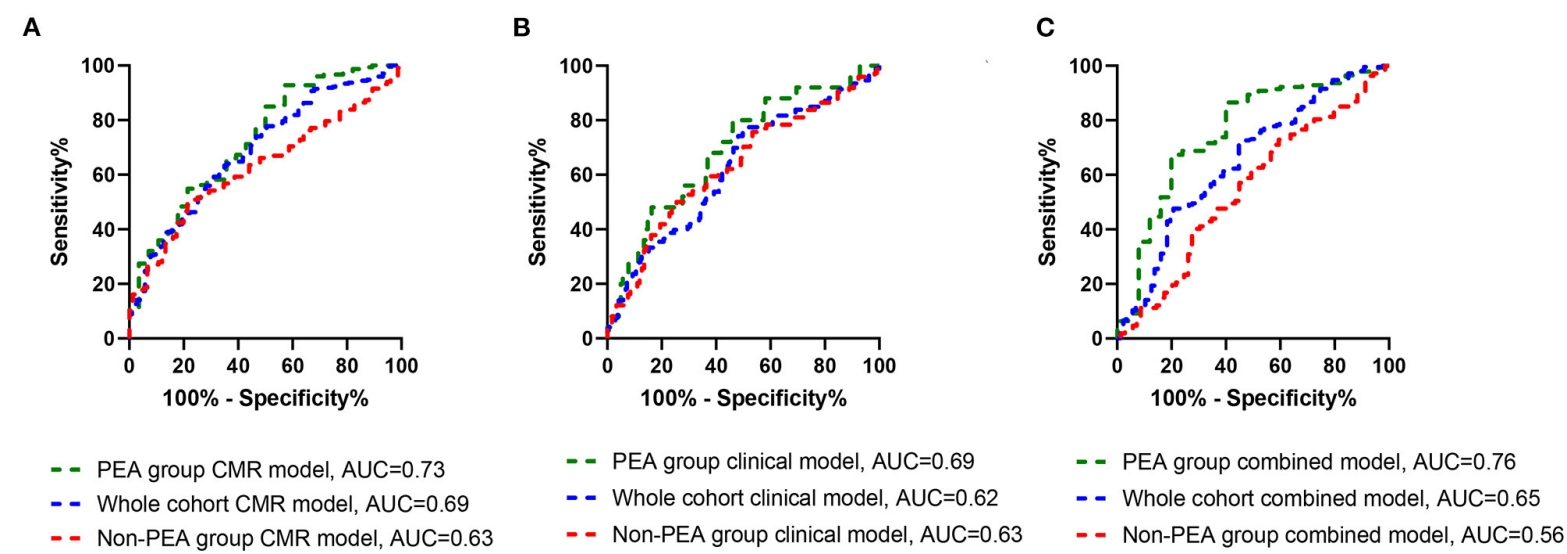
Receiver operating curves for CMR model (green dashes), clinical model (blue dashes) and combined clinical and CMR models (red dashes) in the whole cohort **(A)**, PEA group **(B)**, and in the non-PEA **(C)** showing area under the curve (AUC) for overall mortality.

The accuracy of the multivariate Cox models for the whole, PEA and non-PEA cohorts was tested using ROC analysis and presented as AUC. The prognostic accuracy of the CMR model in the whole cohort (AUC, 0.69, *p* = 0.001), clinical model (AUC, 0.62, *p* < 0.001) and in the combined CMR and clinical model (AUC, 0.65, *p* < 0.001). Sensitivity and specificity of the CMR whole cohort model was 71% (95% CI, 65–76) and 62% (95% CI, 52–72) respectively, clinical model 78% (95% CI, 69–86) and 43% (95% CI, 37–50) and for the combined clinical and CMR model 70% (95% CI, 63–76) and 55% (95% CI, 45–65), respectively (Figure 3A). In the PEA cohort, the prognostic accuracy of the CMR model (AUC, 0.73, *p* < 0.001), clinical model (AUC, 0.69, *p* = 0.002) and in the combined clinical and CMR model (AUC, 0.76, *p* < 0.001). Sensitivity and specificity of the CMR PEA model 80% (95% CI, 61–91) and 53% (95% CI, 46–62), respectively, clinical model 72% (95% CI, 65–79) and 65% (95% CI, 46–79) and for the combined model 80% (95% CI, 61–91) and 53% (95% CI, 46–62), respectively (Figure 3B). The prognostic accuracy of the CMR model in the non-PEA group (AUC, 0.63, *p* = 0.003), clinical model (AUC, 0.63, *p* = 0.003) and for the combined model (AUC, 0.56, *p* = 0.030). The sensitivity and specificity were 65% (95% CI, 56–73) and 60% (95% CI, 50–71), respectively for the CMR model, 60% (95% CI, 48–70) and 58% (95% CI, 50–67), respectively for the clinical model and 60% (95% CI, 48–70) and 58% (95% CI, 50–67), respectively for the combined model (Figure 3C).

### Internal Validation and Bootstrapping

Internal validation was performed using the bootstrap method as previously described. Supplementary Table 3 summarizes the results of the multivariate Cox regression model using the bootstrap method for the whole cohort CMR model. Variables remained significant with no bias after performing 1,000 bootstraps using bias corrected and accelerated confidence intervals. Similarly, variables for CMR prognostic model for the PEA and non-PEA groups remained significant after the bootstrap method (All *p* < 0.050).

## Discussion

To our knowledge this is the first study to demonstrate that CMR has prognostic value in patients with CTEPH. We have also highlighted the importance of considering left heart disease in patients considered for PEA, by showing the prognostic value of LAVI in patients undergoing PEA, whilst confirming the value of established prognostic markers of RV function in patients with CTEPH who do not undergo PEA.

Despite extensive study of the potential value of CMR in patients with PAH, where measures of RV function are strongly prognostic (15, 17, 25, 37–43), there is only limited data on the use of CMR in patients with CTEPH with large cohort studies in CTEPH primarily reporting on the utility of clinical, exercise and haemodynamic measures to predict mortality (24, 34, 44). Studies using CMR in CTEPH (45, 46) have focussed on changes in CMR metrics following PEA. Schoenfeld et al. (45) showed an improvement in RV mass and function 12 days post PEA and Mauritz et al. (46) showed an improvement in RV systolic wall stress 6 months post PEA. Non cardiac focused MRI based techniques such as MR pulmonary perfusion maps and MR pulmonary angiography for the screening of CTEPH and surgical planning have also shown to have diagnostic value (12, 18–20, 47). Combining CMR with MR pulmonary perfusion maps and MR pulmonary angiography provides a potential one-stop shop for comprehensive cardiopulmonary of evaluation of suspected PH with MR imaging, providing information on the likelihood of PH, its severity and potential causes.

In this study we have shown that a number of measures reflecting RV function, including RVESVI%pred which is strongly prognostic in PAH (15, 25) and RVEF (%pred), were predictors of mortality in the whole cohort (Supplementary Table 1) and in those not undergoing PEA (Table 5), at univariate analysis. At multivariate analysis LVSVI %predicted, a measure of global cardiac function was an independent predictor of outcome in the whole cohort whilst RVEF%pred a measure of RV function was an independent predictor of outcome in patients not undergoing PEA. Increases in RV afterload in idiopathic PAH impair RV function resulting in reductions in LVSVI which has been shown to have prognostic value (39) and aid monitoring of treatment (40). In contrast, neither RV size nor RV functional metrics prior to PEA were predictors of mortality in patients undergoing surgery at univariate analysis. In carefully selected patients with surgical disease, PEA can dramatically reduce afterload, normalize pulmonary hemodynamics and improve RV function (45, 48–50) and may explain in significant part why pre-operative measures of RV function are not prognostic in these patients.

A novel finding of this study relates to prognostic value of LA size in patients with CTEPH. LAVI was a significant independent predictor of mortality in the whole cohort and in patients undergoing PEA, highlighting the importance of considering left heart disease when considering the risk and benefits of PEA in CTEPH. Although a LAVI of >41 ml/m^2^ is used as a threshold to identify an enlarged left atrium on transthoracic echocardiography a normal LA size in patients undergoing PEA cannot be used to exclude left heart disease, and this may be unmasked following surgery. PEA results in increased LA and LV filling post-surgery (46) and increases in LAVI of ~20% post PEA have previously been noted and correlated with changes in PVR (51).

Markers of PA stiffness based on using a CMR only measurement method or a combination of CMR and RHC measurement methods were also predictors of mortality at univariate analysis but were not shown to be independent predictors of mortality in the whole, PEA or non-PEA cohorts. Vanderpool et al. (30) reported superior prognostic significance of CMR-derived estimate of RV-arterial coupling Ees/Ea over other invasive and non-invasive measures of RV function in patients referred with pulmonary hypertension. However, no additional prognostic value of a CMR-only measurement of RV–PA coupling over volumetric indices was demonstrated in the present study in patients with CTEPH.

This study also confirms findings from the International CTEPH Registry (52) and our previous work from the ASPIRE Registry regarding the prognostic impact of demographics such as age, comorbidities including malignancy, coronary artery disease and chronic kidney disease, pulmonary hemodynamics and measures of exercise capacity and lung function on outcomes in patients with CTEPH. However, despite inclusion of these prognostic factors MRI metrics still had independent predictive value (Table 5). The prognostic accuracy of the CMR only model was higher compared to the accuracy of the clinical only model and combining both models did not increase the prognostic ability of the model only for the PEA group (Figure 3).

### Study Limitations

This was a single center study with retrospective analysis of prospectively collected data which might have introduced selection or misclassification bias. CMR metrics were corrected for age and sex but not for race, which impacts on CMR metrics (53). Despite identifying LAVI as an independent predictor of outcome in the whole cohort and patients undergoing PEA further work is required to identify thresholds to aid clinical decision making. This study also used CMR as a gold standard technique to assess cardiac volumetric and functional data rather than echocardiography and echocardiography may be able to provide comparative data although this is not answered by this study. Balloon pulmonary angioplasty has only recently been available in the UK and we were unable therefore to provide any data regarding the potential for CMR to assess for outcomes in patients undergoing this intervention.

## Conclusion

CMR metrics reflecting RV function and the presence of left heart disease are of prognostic value in patients with CTEPH. In those undergoing PEA an elevated left atrial volume index predicts a worse outcome highlighting the importance of considering left heart disease in patients considered for PEA. This study also demonstrates the prognostic value of CMR imaging metrics in determining outcome in patients with CTEPH not-undergoing surgery. Whether CMR has a role in serial monitoring of such patients requires further evaluation.

## Data Availability Statement

The raw data supporting the conclusions of this article will be made available by the authors, without undue reservation.

## Ethics Statement

The studies involving human participants were reviewed and approved by University of Sheffield, ref c06/Q2308/8. Written informed consent for participation was not required for this study in accordance with the national legislation and the institutional requirements.

## Author Contributions

YS, AS, and DK conceived the idea for the study and prepared tables and figures. YS, SA, MS, DA, and RL collected and analyzed data for study. YS and AS did statistical analysis. SA, PG, CJ, and SR assisted with the demographic and MRI data. JW, RC, CE, AC, AH, AS, and DK supported this study management and assisted in the writing of the manuscript. AS and DK edited the final manuscript. All authors read and approved the manuscript.

## Funding

This study was funded in part by the Wellcome Trust to AS, grant number: AJS 205188/Z/16/Z.

## Conflict of Interest

The authors declare that the research was conducted in the absence of any commercial or financial relationships that could be construed as a potential conflict of interest.

## Publisher's Note

All claims expressed in this article are solely those of the authors and do not necessarily represent those of their affiliated organizations, or those of the publisher, the editors and the reviewers. Any product that may be evaluated in this article, or claim that may be made by its manufacturer, is not guaranteed or endorsed by the publisher.

## Glossary

CTEPH: chronic thromboembolic pulmonary hypertension
PAH: pulmonary arterial hypertension
mPAP: mean pulmonary artery pressure
RHC: right heart catheter
DLCO: diffusing capacity of the lungs for carbon monoxide
FEV1: forced expiratory volume
FVC: forced vital capacity
ISWD: incremental shuttle walking distance
mPAP: mean pulmonary arterial pressure
mRAP: mean right atrial pressure
PAWP: pulmonary artery wedge pressure
PEA: pulmonary endarterectomy
PVR: pulmonary vascular resistance
SvO2: mixed venous oxygen saturation
%pred: percentage predicted
Ea: arterial load
Ees: right ventricle elastance
LAVI: left atrium volume index
LVEDMI: left ventricle end diastolic mass index
LVEDVI: left ventricle end diastolic volume index
LVEF: left ventricle ejection fraction
LVESVI: left ventricle end systolic volume index
LVSVI: left ventricle stroke volume index
MRI: magnetic resonance imaging
PA: pulmonary artery
PA RAC: pulmonary artery relative area change
RVEDMI: right ventricle end diastolic mass index
RVEDVI: right ventricle end diastolic volume index
RVEF: right ventricle ejection fraction
RVSP: right ventricle systolic pressure
RVESVI: right ventricle end systolic volume index
RVSVI: right ventricle stroke volume index
VMI: ventricular mass index.
