# Supplementary material for: CMR Measures of Left Atrial Volume Index and Right Ventricular Function Have Prognostic Value in Chronic Thromboembolic Pulmonary Hypertension
Source: Front Med (Lausanne). 2022 Mar 14;9:840196. doi: 10.3389/fmed.2022.840196 (PMC8964043; doi:10.3389/fmed.2022.840196)
Supplement: Supplementary file 1 [file Data_Sheet_1.docx]

**Supplementary Table S1**: Patients demographics and results of baseline investigations for survivors and non survivors in the whole cohort

| **Demographics** | **Survivors**  **(n=271)** | **Non survivors**  **(n=104)** | **P value** |
| --- | --- | --- | --- |
| Age, years | 62 (14) | 69 (12) | <0.001 |
| Female, n (%) | 135 (36) | 50 (13) | <0.001 |
| BMI, Kg/m^2^ | 29 (6) | 28 (6) | 0.055 |
| PEA, n (%) | 153 (40) | 28 (7) | <0.001 |
| ISWD, m | 284 (210) | 154 (132) | <0.001 |
| ***Comorbidities, n (%)*** | | | |
| Malignancy | 24 (9) | 28 (27) | 0.001 |
| CAD | 21 (8) | 24 (23) | <0.001 |
| Left Heart Failure | 11 (4) | 8 (8) | 0.150 |
| CKD | 15 (6) | 12 (12) | 0.041 |
| COPD | 20 (7) | 15 (14) | 0.036 |
| AF | 28 (10) | 18 (17) | 0.065 |
| CVA | 21 (8) | 5 (5) | 0.315 |
| ***Cardiac MR metrics*** | | | |
| RVEDVI, ml/m^2^ | 82 (32) | 99 (30) | 0.041 |
| RVEDVI %pred | 110 (41) | 125 (48) | 0.003 |
| RVESVI, ml/m^2^ | 50 (27) | 59 (28) | 0.007 |
| RVESVI %pred | 211 (116) | 268 (138) | <0.001 |
| RVEF, % | 41 (14) | 36 (11) | 0.004 |
| RVEF %pred | 60 (21) | 53 (17) | 0.001 |
| RVSVI, ml/m^2^ | 32 (13) | 31 (12) | 0.562 |
| RVSVI %pred | 64 (26) | 62 (23) | 0.709 |
| RVEDMI, g/m^2^ | 20 (10) | 24 (12) | 0.002 |
| RVEDMI %pred | 70 (33) | 84 (42) | 0.001 |
| LVEDVI, ml/m^2^ | 58 (16) | 54 (14) | 0.025 |
| LVEDVI %pred | 78 (21) | 74 (20) | 0.127 |
| LVESVI, ml/m^2^ | 20 (8) | 20 (11) | 0.708 |
| LVESVI %pred | 82 (33) | 87 (49) | 0.263 |
| LVEF, % | 66 (10) | 64 (12) | 0.061 |
| LVEF %pred | 98 (14) | 94 (17) | 0.027 |
| LVSVI, ml/m^2^ | 38 (11) | 34 (9) | <0.001 |
| LVSVI %pred | 77 (23) | 69 (19) | 0.002 |
| LVEDMI, g/m^2^ | 48 (11) | 50 (12) | 0.142 |
| LVEDMI %pred | 70 (14) | 73 (17) | 0.084 |
| Systolic septal angle, degrees | 165 (22) | 168 (22) | 0.162 |
| Diastolic septal angle, degrees | 142 (9) | 144 (10) | 0.031 |
| LAVI, ml/m^2^ | 34 (13) | 39 (23) | 0.002 |
| VMI, % | 0.44 (0.20) | 0.50 (0.20) | 0.011 |
| Positive aortic flow, L/min/m^2^ | 5.7 (2) | 5.6 (2) | 0.611 |
| PA flow positive index, L/min/m^2^ | 5 (2) | 5 (2) | 0.288 |
| PA flow negative index, L/min/m^2^ | 15 (9) | 18 (12) | 0.002 |
| Systolic PA area, mm^2^ | 942 (223) | 986 (259) | 0.109 |
| Diastolic PA area, mm^2^ | 848 (200) | 904 (240) | 0.024 |
| ***Right heart catheter metrics*** | | | |
| PA SBP, mmHg | 74 (23) | 78 (21) | 0.189 |
| PA DBP, mmHg | 25 (8) | 27 (8) | 0.018 |
| mPAP, mmHg | 43 (12) | 46 (12) | 0.074 |
| mRAP, mmHg | 10 (5) | 12 (5) | 0.001 |
| RVSP, mmHg | 71 (23) | 76 (21) | 0.104 |
| PCWP, mmHg | 12 (4) | 13 (4) | 0.032 |
| PVR, dyne.s.cm^-5^ | 558 (353) | 663 (399) | 0.036 |
| SVR, WU | 1700 (668) | 1752 (714) | 0.536 |
| CO, L/min | 5 (1) | 4 (1) | 0.027 |
| CI, L/min/m^2^ | 2.5 (0.7) | 2.3 (0.7) | 0.048 |
| SaO2, % | 93 (4) | 92 (4) | 0.162 |
| SvO2, % | 64 (7) | 59 (8) | <0.001 |
| ***Lung function tests*** | | | |
| FEV1, L | 2.32 (0.81) | 1.83 (0.60) | <0.001 |
| FVC, L | 3.34 (1.10) | 2.91 (0.90) | 0.003 |
| DLCO %pred | 54 (23) | 44 (12) | <0.001 |
| ***PA stiffness and RV-PA coupling metrics*** | | | |
| PA RAC, ratio | 12 (11) | 10 (7) | 0.088 |
| Ees, mmHg/ml/m^2^ | 1 (0.5) | 0.9 (0.4) | 0.076 |
| Ea, mmHg/ml/m^2^ | 0.9 (0.6) | 1 (0.7) | 0.113 |
| Ees/Ea ratio | 2 (1) | 1 (1) | 0.023 |
| MRI Ees/Ea ratio | 0.41 (0.1) | 0.36 (0.1) | 0.003 |
| PA distensibility, (ΔV/V)/ΔP | 0.19 (0.2) | 0.20 (0.2) | 0.826 |

**Supplementary Table S1:** Patients demographics and results of baseline investigations for survivors and non survivors in the whole cohort. Data is presented as mean (SD) unless otherwise stated. For abbreviation list see legend for tables 1 and 2.

**Supplementary Table S2**: Univariate Cox Proportional Hazard regression analysis in the whole CTEPH cohort (data shown where p<0.20)

| **Covariate** | **Univariate Hazard Ratio** | **Scaled Univariate Hazard Ratio** | **P value** |
| --- | --- | --- | --- |
| Age, years | 1.041 (1.023-1.059) | 1.053 (1.019-1.060) | <0.001 |
| ISWD, m | 0.996 (0.995-0.997) | 0.885 (0.880-0.999) | <0.001 |
| PEA | 0.304 (0.203-0.470) | 0.354 (0.233-0.500) | <0.001 |
| ***Comorbidities, n*** | | | |
| Malignancy | 2.276 (1.438-3.601) |  | <0.001 |
| CAD | 3.078 (1.942-4.877) |  | <0.001 |
| Left Heart Failure | 1.738 (0.842-3.587) |  | 0.135 |
| CKD | 1.766 (0.967-3.228) |  | 0.064 |
| COPD | 1.880 (1.085-3.257) |  | 0.024 |
| ***Cardiac MR metrics*** | | | |
| RVEDVI %pred | 1.006 (1.002-1.010) | 1.336 (1.140-1.580) | 0.002 |
| RVESVI %pred | 1.003 (1.001-1.004) | 1.417 (1.231-1.642) | <0.001 |
| RVEF %pred | 0.982 (0.973-0.992) | 0.631 (0.516-0.772) | <0.001 |
| RVEDMI %pred | 1.006 (1.002-1.010) | 1.234 (1.056-1.441) | 0.008 |
| LVEDVI %pred | 0.992 (0.982-1.001) | 0.806 (0.662-0.981) | 0.096 |
| LVEF %pred | 0.985 (0.973-0.996) | 0.791 (0.661-0.945) | 0.011 |
| LVSVI %pred | 0.985 (0.977-0.994) | 0.669 (0.590-0.995) | 0.001 |
| LVEDMI %pred | 1.009 (0.996-1.022) | 1.178 (0.975-1.423) | 0.184 |
| LAVI, ml/m^2^ | 1.010 (1.002-1.018) | 1.181 (1.028-1.356) | 0.010 |
| ***Right heart catheter metrics*** | | | |
| mPAP, mmHg | 1.016 (0.999-1.033) | 1.251 (1.021-1.534) | 0.060 |
| mRAP, mmHg | 1.059 (1.024-1.097) | 1.307 (1.098-1.555) | 0.001 |
| PVR, dyne.s.cm^-5^ | 1.001 (1.000-1.001) | 1.296 (1.080-1.556) | 0.005 |
| CO, L/min | 0.784 (0.669-0.919) | 0.701 (0.556-0.884) | 0.003 |
| SvO2, % | 0.925 (0.902-0.948) | 0.565 (0.472-0.677) | <0.001 |
| ***Lung function tests*** | | | |
| FEV1 %pred | 0.533 (0.390-0.728) | 0.641 (0.498-0.825) | <0.001 |
| DLCO %pred | 0.664 (0.575-0.767) | 0.479 (0.361-0.635) | <0.001 |
| ***PA stiffness and RV-PA coupling metrics*** | | | |
| PA RAC, ratio | 0.984 (0.965-1.004) | 0.852 (0.692-1.050) | 0.117 |
| Ees, mmHg/ml/m^2^ | 0.641 (0.392-1.049) | 0.757 (0.601-0.954) | 0.077 |
| Ea, mmHg/ml/m^2^ | 1.299 (0.951-1.776) | 1.254 (1.041-1.510) | 0.101 |
| Ees/Ea ratio | 0.770 (0.610-0.971) | 0.649 (0.472-0.893) | 0.027 |
| MRI Ees/Ea ratio | 0.094 (0.022-0.392) | 0.652 (0.530-0.802) | 0.001 |

**Supplementary Table S2:** Univariate Cox Proportional Hazard regression analysis in the whole CTEPH (data shown where p<0.20). For abbreviations see legend for tables 1 and 2.

**Supplementary Table S3**: Multivariate Cardiac MR Metrics and combined models in the whole CTEPH cohort following bootstrap

| **Covariate** | **B** | **Bias** | **SE** | **P value** | **BCA 95% confidence interval** |
| --- | --- | --- | --- | --- | --- |
| ***Cardiac MR Model*** | | | | | |
| RVESVI %pred | 0.002 | 0.000 | 0.001 | 0.003 | 0.001-0.004 |
| LVSVI %pred | -0.017 | 0.000 | 0.004 | 0.001 | -0.025- -0.009 |
| LAVI, ml/m^2^ | 0.013 | 0.003 | 0.006 | 0.013 | 0.008-0.035 |
| ***Combined Cardiac MR and Clinical Model*** | | | | | |
| ISWD, m | -0.003 | 0.000 | 0.001 | 0.005 | -0.007- -0.001 |
| PEA, n | -1.122 | -0.163 | 0.365 | 0.002 | -1.627- -0.925 |
| CAD, n | 0.893 | 0.018 | 0.355 | 0.003 | 0.217-1.676 |
| Malignancy, n | 0.733 | 0.047 | 0.376 | 0.034 | -0.088-1.643 |
| CKD, n | 0.729 | -0.005 | 0.391 | 0.033 | -0.099-1.525 |
| SvO2, % | -0.045 | -0.003 | 0.028 | 0.040 | -0.101-0.003 |
| LVSVI %pred | -0.048 | 0.000 | 0.025 | 0.018 | -0.098-0.005 |
| LAVI, ml/m^2^ | 0.014 | 0.003 | 0.011 | 0.030 | -0.008-0.055 |

**Supplementary Table S3**: Multivariate cardiac MR metrics and combined cox regression models in the whole cohort following bootstrapping. B, beta coefficient; SE, standard error; BCA, bias corrected accelerated. For other abbreviations see legend for tables 1 and 2.
